# Dual roles of inflammatory programmed cell death in cancer: insights into pyroptosis and necroptosis

**DOI:** 10.3389/fphar.2024.1446486

**Published:** 2024-08-27

**Authors:** Shuai Wang, Huanhuan He, Lailiang Qu, Qianhe Shen, Yihang Dai

**Affiliations:** ^1^ Collage of Medicine, Xinyang Normal University, Xinyang, China; ^2^ State Key Laboratory of Biocontrol, Guangdong Provincial Key Laboratory of Plant Resources and Southern Marine Science and Engineering Guangdong Laboratory (Zhuhai), School of Life Sciences, Sun Yat-sen University, Guangzhou, China

**Keywords:** pyroptosis, necroptosis, cancer, inflammation, anti-cancer drugs

## Abstract

Programmed cell death (PCD) is essential for cellular homeostasis and defense against infections, with inflammatory forms like pyroptosis and necroptosis playing significant roles in cancer. Pyroptosis, mediated by caspases and gasdermin proteins, leads to cell lysis and inflammatory cytokine release. It has been implicated in various diseases, including cancer, where it can either suppress tumor growth or promote tumor progression through chronic inflammation. Necroptosis, involving RIPK1, RIPK3, and MLKL, serves as a backup mechanism when apoptosis is inhibited. In cancer, necroptosis can enhance immune responses or contribute to tumor progression. Both pathways have dual roles in cancer, acting as tumor suppressors or promoting a pro-tumorigenic environment depending on the context. This review explores the molecular mechanisms of pyroptosis and necroptosis, their roles in different cancers, and their potential as therapeutic targets. Understanding the context-dependent effects of these pathways is crucial for developing effective cancer therapies.

## Graphical Abstract

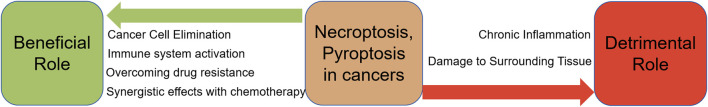

## 1 Introduction

Programmed cell death (PCD) is a fundamental biological process crucial for maintaining cellular homeostasis, eliminating damaged cells, and defending against infections. PCD can be categorized into inflammatory and non-inflammatory pathways. The inflammatory forms of PCD include pyroptosis and necroptosis, which play significant roles in various pathological conditions, including cancer, infections, and inflammatory diseases. Unlike non-inflammatory forms such as apoptosis, autophagy, and phagoptosis, inflammatory cell death is characterized by the release of pro-inflammatory cytokines and cellular contents that can provoke strong immune responses. Understanding the molecular mechanisms and roles of these inflammatory cell death pathways is essential for developing novel therapeutic strategies.

Pyroptosis was initially observed in macrophages infected with *Shigella* and *Salmonella* (Boise and Collins, 2001), and has since been implicated in a variety of diseases, including cancer. Pyroptosis is a form of programmed cell death distinguished by its reliance on caspases, particularly caspase-1, -4, -5, and -11, leading to the cleavage of gasdermin proteins that form pores in the cell membrane (Shi et al., 2014). This process results in cell lysis and the release of inflammatory cytokines such as IL-1β and IL-18. The discovery of non-classical pyroptosis pathways involving caspase-3 and caspase-8, as well as the roles of other gasdermin family members like gasdermin E (GSDME) and gasdermin C (GSDMC), has expanded our understanding of this complex cell death mechanism (Wang et al., 2017).

Necroptosis, another form of inflammatory programmed cell death, is mediated by receptor-interacting protein kinases-1/-3 (RIPK1 and RIPK3) and mixed lineage kinase domain-like protein (MLKL). Upon activation by death receptors or pathogen recognition receptors, RIPK1 interacts with RIPK3 to form a necrosome, which subsequently phosphorylates MLKL. The phosphorylated MLKL translocates to the plasma membrane, forming disruptive pores that lead to cell swelling, membrane rupture, and the release of damage-associated molecular patterns (DAMPs). Necroptosis is often a backup mechanism when apoptosis is inhibited, playing a crucial role in immune responses and disease pathogenesis.

Inflammatory programmed cell death, encompassing both pyroptosis and necroptosis, contributes significantly to cancer biology. In the tumor microenvironment, these pathways can either suppress tumor growth by eliminating malignant cells or promote tumor progression through chronic inflammation and immune evasion. For instance, in non-small cell lung cancer (NSCLC), elevated gasdermin D (GSDMD) expression has been linked to increased tumor invasion and metastasis, while the induction of pyroptosis can lead to cancer cell apoptosis. Similarly, in breast cancer, gasdermin B (GSDMB) expression correlates with enhanced cell invasion and poor prognosis. Conversely, activating pyroptosis or necroptosis pathways in colorectal cancer cells has shown potential in overcoming chemoresistance and inhibiting tumor growth.

The dual roles of pyroptosis and necroptosis in cancer underscore the complexity of these pathways. While they can act as tumor suppressors by triggering inflammatory cell death, they can also facilitate a pro-tumorigenic environment through the release of inflammatory mediators. This paradox highlights the importance of precise modulation of these pathways in cancer therapy. Research efforts are increasingly focused on understanding the context-dependent effects of pyroptosis and necroptosis, aiming to harness their therapeutic potential while mitigating adverse inflammatory responses.

## 2 Mechanisms of pyroptosis

### 2.1 Inflammasome-mediated canonical pyroptosis pathway

The inflammasome is a multiprotein complex that includes major types such as NLRP1, NLRP3, NLRC4, and AIM2. Upon exposure to exogenous or endogenous microbial infections, stimulatory factors, or damage signals, pattern recognition receptors (PRRs) and DAMPs recognize and bind to their respective ligands, forming the inflammasome complex. This complex subsequently promotes the maturation of pro-caspase-1 into active caspase-1. Caspase-1 then processes various inflammatory cytokines (e.g., IL-1β, IL-6, and IL-18) into their mature forms (Wang et al., 2024). Concurrently, caspase-1 cleaves GSDMD into its active form, GSDMD-N, which translocates to the cell membrane to form pores. The formation of these pores facilitates the rapid release of mature inflammatory cytokines into the extracellular space, triggering an amplified inflammatory response in the surrounding environment, leading to cell swelling, bubbling, and eventual death. This pathway, dependent on inflammasomes, caspase-1, and GSDMD, is known as the classical pyroptosis pathway.

### 2.2 Non-canonical pyroptosis pathway

Recent studies have demonstrated that some forms of pyroptosis do not rely on the inflammasome. Lipopolysaccharides (LPS) can directly bind to and activate caspase-4, -5, and -11, promoting the cleavage and maturation of GSDMD, thus inducing the non-canonical pyroptosis pathway (Shi et al., 2014). For instance, the *Yersinia* effector protein YopJ inhibits the activity of TGF-β-activated kinase 1 (TAK1), leading to the caspase-8-mediated cleavage of GSDMD (Orning et al., 2018). Additionally, granzyme A (GZMA), secreted by cytotoxic lymphocytes, can induce pyroptosis through the cleavage of GSDMB (Zhou et al., 2020), and neutrophil elastase (ELANE) can induce pyroptosis via GSDMD cleavage (Kambara et al., 2018). These findings suggest that the pathways leading to pyroptosis are complex, with an increasing number of non-inflammasome-mediated pyroptosis mechanisms being discovered.

## 3 Mechanisms of necroptosis

Necroptosis can be activated by factors such as the tumor necrosis factor receptor (TNFR) superfamily, T-cell receptors, pattern recognition receptors, and oxidative stress (Lalaoui et al., 2015). Taking the classic TNF/TNFR signaling pathway as an example, the binding of TNF to TNFR1 induces the formation of a membrane complex. This complex includes the inhibitor of apoptosis proteins (IAP) family members cIAP1 and cIAP2, which ubiquitinate RIPK1. The ubiquitination of RIPK1 promotes the activation of nuclear factor kappa-B (NF-κB) and mitogen-activated protein kinase (MAPK) signaling pathways, leading to the transcription of downstream genes, including FLICE-like inhibitory protein (c-FLIP), which inhibits the activity of caspase-8 (Wright et al., 2007; Bertrand et al., 2008). Under normal conditions, RIPK1 and RIPK3 are cleaved and inactivated by caspase-8. However, during necroptosis, the inactivation of caspase-8 prevents the cleavage of RIPK1 and RIPK3. Subsequently, RIPK1 and RIPK3 undergo phosphorylation to form the necrosome, which phosphorylates and oligomerizes MLKL. The oligomerized MLKL complex translocates to the cell membrane to form pores, initiating necroptosis (Cho et al., 2009; Sun et al., 2012; Murphy et al., 2013).

Recent studies have revealed that, beyond the classical RIPK3-MLKL signaling pathway, RIPK3 can also induce necroptosis through phosphoglycerate mutase 5 (PGAM5) and calcium/calmodulin-dependent protein kinase II (CAMK II). PGAM5 has two isoforms, PGAM5L and PGAM5S. Upon activation, RIPK3 phosphorylates PGAM5L, which then binds to PGAM5S on the mitochondrial membrane. The PGAM5L/PGAM5S complex induces mitochondrial fission by dephosphorylating dynamin-related protein 1, leading to necroptosis.

## 4 Lung cancer

### 4.1 Necroptosis and lung cancer

Lung cancer encompasses various types with complex etiologies, and recent research into necroptosis has opened new avenues for its treatment. Studies have shown that RIPK3 expression is significantly lower in NSCLC patients, correlating with poor chemotherapy outcomes (Lim et al., 2021). Ectopic expression of RIPK3 markedly increases the sensitivity of lung cancer cells to chemotherapeutic agents such as cisplatin, etoposide, vincristine, and adriamycin (Wang Q. et al., 2020). Traditionally, cisplatin was believed to exert its anticancer effects by inducing apoptosis in lung cancer cells. However, recent findings suggest that cisplatin also mediates its effects through RIPK1-RIPK3-MLKL-dependent necroptosis (Jing et al., 2018). Another study indicates that the compound HS-173 induces necrosis in lung cancer cells by enhancing RIPK3 expression and activating the RIPK3/MLKL signaling pathway (Park et al., 2019). Furthermore, 2-methoxy-6-acetyl-7-methyljuglone (MAM) exerts anticancer effects by targeting RIPK1, causing oxidative stress, and inducing necrosis in A549 and H1299 lung cancer cells (Sun et al., 2019). Additionally, tanshinol A has been shown to mediate necroptosis in lung cancer cells via MLKL, independent of RIPK1 and RIPK3 activation, by directly inducing MLKL phosphorylation and oligomerization (Liu et al., 2020).

### 4.2 Pyroptosis and lung cancer

Research has demonstrated that GSDMD expression is significantly elevated in NSCLC and is associated with tumor cell invasion and metastasis (Gao et al., 2018), knockout of GSDMD, while not leading to pyroptosis, induces apoptosis when the pyroptotic signaling pathway is activated. Furthermore, the active component of Paris polyphylla, polyphyllin VI, induces pyroptosis in NSCLC cells by activating the NLRP3-Caspase1-GSDMD signaling pathway (Teng et al., 2020). Cucurbitacin B also induces pyroptosis in NSCLC cells by directly binding to Toll-like receptor 4 (TLR4) and activating NLRP3 and GSDMD (Yuan et al., 2021). Moreover, the classical chemotherapeutic agent cisplatin can induce pyroptosis in A549 lung cancer cells by activating Caspase-3, which cleaves GSDME to produce GSDME-N (Zhang CC. et al., 2019). The oncogenic LncRNA-XIST regulates the proliferation, invasion, and migration of NSCLC cells (Sun et al., 2017; Wang X. et al., 2018; Liu A. et al., 2019; Zhang J. et al., 2019). Knockdown of LncRNA-XIST significantly elevates reactive oxygen species levels in A549 cells, inducing pyroptosis in cancer cells (Cui et al., 2019; Liu J. et al., 2019).

Contrary to the aforementioned findings, some studies have reported different outcomes. Wang et al. (2016) demonstrated that LPS combined with ATP enhances the proliferation and migration of A549 cells by activating the NLRP3 inflammasome. Salidroside, on the other hand, reduces LPS-induced inflammasome activation, thereby inhibiting the proliferation and migration of A549 cells (Ma et al., 2021). Liang et al. (2020) found that tumor-derived exosomal TRIM59 promotes lung cancer progression by modulating macrophages to a tumor-promoting phenotype through ABHD5 proteasomal degradation, which in turn activates the NLRP3 inflammasome signaling pathway and promotes IL-1β secretion.

## 5 Colorectal cancer

### 5.1 Necroptosis and colorectal cancer

Colorectal cancer (CRC) ranks as the fourth most common cancer worldwide, claiming nearly 700,000 lives annually. Investigating necroptosis and pyroptosis offers promising avenues for discovering new therapeutic strategies for CRC. Studies have demonstrated that bufogenin mediates necroptosis through the upregulation of RIPK3 and phosphorylation of MLKL, effectively inhibiting CRC development and metastasis (Han et al., 2018). Additionally, the small molecule GDC-0326 induces necroptosis in CRC cells by modulating RIPK1 and RIPK3. When combined with 5-fluorouracil (5-FU), GDC-0326 significantly overcomes 5-FU resistance in CRC treatment, enhancing its anticancer efficacy (Zhang et al., 2021). SMYD2, a histone methyltransferase, is implicated in various cancers. Research indicates that SMYD2 expression is significantly elevated in both human and murine colorectal cancer tissues. The absence of SMYD2 sensitizes colon tumor cells to TNF-induced apoptosis and necroptosis. Further studies reveal that SMYD2 targets RIPK1, inhibiting its phosphorylation and thereby preventing necroptosis (Yu et al., 2022).

However, conflicting views exist regarding the role of necroptosis in CRC. Some studies suggest that PGAM5, downstream of RIPK3, is upregulated in CRC, contributing to cancer progression by affecting NADPH production and lipid metabolism (Zhu et al., 2020). Elevated RIPK3 expression is also observed in mouse models of colitis-associated cancer and human CRC. RIPK3 knockout mice are protected against dextran sulfate sodium (DSS)-induced colitis-associated cancer (Liu ZY. et al., 2019). Since necroptosis is associated with increased inflammation, which is a risk factor for colitis and subsequently CRC, excessive necroptosis may accelerate cancer progression through heightened inflammatory responses.

### 5.2 Pyroptosis and colorectal cancer

Research indicates that the antitumor drug lobaplatin mediates ROS/JNK/Bax signaling, inducing pyroptosis via caspase-3/-9-dependent GSDME cleavage, thereby exerting its antitumor effects (Yu et al., 2019). Notably, even when GSDME is knocked out, lobaplatin still induces tumor cell death through apoptosis, demonstrating its robust antitumor activity (Yu et al., 2019). Multiple studies have shown that inflammasome-mediated pyroptosis can suppress tumorigenesis. Mice deficient in NLRP1, NLRP3, NLRC4, AIM2, or pyrin inflammasomes exhibit significantly higher tumor incidence (Allen et al., 2010; Hu et al., 2010; Williams et al., 2015; Wilson et al., 2015; Sharma et al., 2018). Additionally, NLRP1 expression is markedly lower in colorectal cancer tissues compared to normal tissues (Chen et al., 2015). The antitumor drug 5-aza-2-deoxycytidine inhibits CRC progression by restoring NLRP1 expression, suggesting that NLRP1 is a potential therapeutic target for CRC (Chen et al., 2015).

The intestinal epithelial barrier is crucial for maintaining gut homeostasis. Its impairment can lead to immune dysregulation and inflammatory responses, accelerating CRC progression. Bauer et al. (2010) found that NLRP3 plays a critical role in DSS-induced colitis. NLRP3 knockout mice exhibit significantly less severe colitis compared to wild-type mice after DSS administration. Moreover, the absence of IL-18 and its receptor protects mice from DSS-induced colitis (Nowarski et al., 2015). Contrarily, other studies report that NLRP1b, NLRP3, and pyrin promote epithelial barrier regeneration and prevent CRC progression in early stages by secreting IL-18 (Allen et al., 2010; Dupaul-Chicoine et al., 2010; Zaki et al., 2010). ASC and caspase-1 knockout mice are also more susceptible to DSS-induced colitis and colitis-associated colorectal cancer (Dupaul-Chicoine et al., 2010; Hirota et al., 2011). These findings suggest that inflammasomes maintain gut homeostasis, reducing inflammation and CRC incidence. The discrepancy in conclusions may arise because inflammasome-mediated IL-18 production aids epithelial barrier regeneration and reduces inflammation in early CRC stages, while excessive cytokine release in advanced stages may accelerate cancer progression. Therefore, pyroptosis plays a significant role in CRC development and treatment, though its mechanisms warrant further investigation.

## 6 Breast cancer

### 6.1 Necroptosis and breast cancer

Breast cancer, a malignant tumor originating from the uncontrolled proliferation and malignancy of breast epithelial cells, is the most common malignancy worldwide (Karamanou et al., 2020). The etiology of breast cancer is complex, involving immutable genetic factors and modifiable factors such as obesity and exogenous hormones. Recent research into necroptosis and pyroptosis has provided new insights for breast cancer treatment. Studies have identified Z-DNA binding protein 1 (ZBP1) as a crucial regulator of necroptosis in breast cancer cells. The absence of ZBP1 disrupts necroptosis during tumor development, and glucose deprivation triggers ZBP1-dependent necroptosis (Baik et al., 2021). Additionally, 5-fluorouracil (5-FU) induces necroptosis in breast cancer cells by activating RIPK1 when caspases are inhibited (Zhang et al., 2018). Shikonin inhibits the proliferation of human triple-negative breast cancer cells, with a dose-dependent increase in RIPK1 expression (Yuan et al., 2021). *In vitro* studies have shown that the combination of Goniothalamin and the caspase inhibitor Z-VAD-FMK activates the RIPK1-RIPK3-MLKL pathway, inducing necroptosis in human breast cancer MDA-MB-231 cells (Khaw-On et al., 2019).

### 6.2 Pyroptosis and breast cancer

Pyroptosis is also closely related to the development and treatment of breast cancer. Various antibiotics can induce pyroptosis in breast cancer cells by upregulating PD-L1 and GSDMC expression and activating Caspase-8 (Hou et al., 2020). Docosahexaenoic acid (DHA) exerts anticancer effects by activating Caspase-1 and GSDMD to induce pyroptosis in breast cancer cells, an effect that can be abolished by Caspase-1 inhibitors (Pizato et al., 2018). Human umbilical cord mesenchymal stem cell-conditioned medium (hUCMSC-CM) can mediate pyroptosis in human breast cancer cells (MCF7) through both NLRP1 and Caspase-4 pathways. When Caspase-4 expression is knocked down, hUCMSC-CM induces pyroptosis via the classical NLRP1 pathway; conversely, knocking down NLRP1 leads to pyroptosis through the non-classical Caspase-4 pathway (Jiao et al., 2020). On the other hand, inflammasomes and the inflammation they mediate are closely associated with breast cancer progression. GSDMB is highly expressed in breast cancer cells and is linked to cancer cell invasion and metastasis (Hergueta-Redondo et al., 2014). The activation of inflammasomes and pyroptosis-derived IL-1β is essential for the proliferation, invasion, and migration of breast cancer cells (Voronov et al., 2003). Tumor growth and metastasis are significantly reduced in NLRP3 knockout mice, with primary and metastatic tumors associated with elevated IL-1β levels. Blocking IL-1β with an IL-1R antagonist inhibits tumor growth and metastasis in breast cancer models (Guo et al., 2016). Obesity increases the risk of breast cancer, and studies by Kolb et al. indicate that the tumor microenvironment in obesity induces tumor-infiltrating myeloid cells via the NLRC4 inflammasome, which activates IL-1β. IL-1β, in turn, drives breast cancer development through adipocyte-mediated expression of vascular endothelial growth factor A (VEGFA) and angiogenesis (Kolb et al., 2016).

## 7 Liver cancer

### 7.1 Necroptosis and liver cancer

A research investigates the pivotal role of necroptosis in both human and murine models of non-alcoholic steatohepatitis (NASH), shedding light on its contribution to hepatocyte death and inflammation (Afonso et al., 2015). Similarly, Gautheron et al. (2014) uncover a positive feedback loop between RIPK3 and JNK in driving hepatocyte death and liver inflammation in NASH. Moreover, Schneider et al. (2017) elucidate the tumor-suppressive function of receptor-interacting protein kinase 1 (RIPK1) in hepatocellular carcinoma (HCC), inhibiting a TNF receptor-associated factor 2 (TRAF2)-dependent pathway to liver cancer. Although another study focuses on the regulatory role of APF long non-coding RNA (lncRNA) in autophagy and myocardial infarction, it underscores the broader relevance of non-coding RNAs in cellular processes (Wang et al., 2015). Collectively, these findings underscore the intricate involvement of necroptosis in liver pathophysiology, highlighting its potential as a therapeutic target for managing liver diseases, including NASH and HCC.

### 7.2 Pyroptosis and liver cancer

Research has demonstrated that NLRP3 expression is significantly downregulated or even completely lost in HCC, with this deficiency promoting cancer progression (Wei et al., 2014). The same research group later discovered that 17β-estradiol (E2) can inhibit the further development of HCC by upregulating NLRP3 (Wei et al., 2015). This upregulation activates NLRP3-mediated pyroptosis, ultimately leading to cancer cell death and exerting anticancer effects (Wei et al., 2019). On the other hand, some studies suggest that excessive activation of NLRP3 is closely associated with hepatitis, liver fibrosis, and cirrhosis (Wree et al., 2014; Mridha et al., 2017; Xie et al., 2020), all of which increase the risk of liver cancer. Additionally, knocking out interleukin-1 receptor-associated kinase 1 (IRAK1) can inhibit the progression of HCC by blocking the MAPKs/IL-1β pathway through NLRP3 suppression (Chen et al., 2020). Furthermore, anisodamine inhibits HCC cell growth, induces apoptosis, and modulates inflammatory cytokine levels by suppressing NLRP3 activation (Li et al., 2020). Direct targeting of the NLRP3 inflammasome also inhibits HCC proliferation, metastasis, and invasion (Fan et al., 2014). These studies collectively suggest that inflammasomes and pyroptosis play critical roles in the development and treatment of HCC. However, the conclusions are not entirely consistent, and the precise mechanisms of action require further elucidation.

## 8 Gastric cancer

### 8.1 Necroptosis and gastric cancer

Necroptosis-related genes (NRGs) have been found to be dysregulated in gastric cancer, suggesting their potential as biomarkers for early diagnosis and prognosis. For instance, studies have identified key NRGs such as CCT6A and FAP, which can effectively predict early Gastric Cancer (GC) and its prognosis. These genes are linked to the response to immunotherapy and immune checkpoint inhibitors, indicating their relevance in personalized cancer treatment strategies. The immunogenic nature of necroptosis could be exploited to enhance anti-cancer immune responses in GC patients (Gong et al., 2019).

A study identified a set of necroptosis-related genes and developed a risk score model to predict prognosis and therapeutic potential in GC patients. This model revealed that patients with higher risk scores had worse clinical outcomes and lower immune cell infiltration, suggesting a correlation between necroptosis and immune response in GC (Wu et al., 2023). Furthermore, necroptosis was found to be triggered by the TNF pathway in myeloid cells, altering the glycolysis pathway and impacting cell functions such as proliferation and migration (Wu et al., 2023).

Additionally, another study highlighted the role of necroptosis-related lncRNAs in distinguishing between “cold” and “hot” tumors in GC. These lncRNAs were associated with different immune infiltration patterns and clinical outcomes, indicating that necroptosis might influence the tumor microenvironment and response to immunotherapy (Zhao et al., 2021).

### 8.2 Pyroptosis and gastric cancer

Multiple subtypes of the GSDM protein family are closely associated with gastric cancer, though their roles differ. GSDMA, GSDMC, and GSDMD are downregulated in gastric cancer tissues or models and may act as tumor suppressor genes (Saeki et al., 2009). The downregulation of GSDMD activates the STAT3 and PI3K/PKB signaling pathways and accelerates the S/G2 phase transition by regulating cell cycle-related proteins, significantly promoting tumor proliferation both *in vitro* and *in vivo* (Wang WJ. et al., 2018). In contrast, GSDMB may function as an oncogene in gastric cancer. It is either not expressed or expressed at very low levels in normal gastric tissue, moderately expressed in precancerous tissue, and highly expressed in cancerous tissue, indicating its potential involvement in gastric cancer development (Komiyama et al., 2010).

*Helicobacter pylori* infection is closely related to various gastric diseases, including gastric cancer (Graham, 2014; Hardbower et al., 2014), primarily through the induction of chronic inflammation that leads to chronic gastritis and subsequent gastric cancer. Research by Kim et al. (2013) has shown that dendritic cells infected with *H. pylori* synergistically produce IL-1β via the TLR2/NOD2 and NLRP3 pathways. Subsequently, Semper et al. (2014) confirmed that *H. pylori* activates NLRP3 to mediate IL-1β production. The cytotoxin-associated gene A (CagA), a major virulence factor of *H. pylori*, mediates the invasion and migration of gastric cancer cells through the NLRP3 pathway (Zhang X. et al., 2020).

Moreover, NLRP3 itself is closely associated with gastric cancer. NLRP3 is significantly upregulated in gastric cancer tissues, mediating the secretion of IL-1β, which promotes epithelial cell proliferation and tumor development. *H. pylori* can decrease the expression of miR-22, an NLRP3 inhibitor, thereby enhancing NLRP3 expression (Li et al., 2018). However, there are differing views on the role of NLRP3 in gastric cancer. Diosbulbin-B effectively increases the sensitivity of gastric cancer cells to the chemotherapeutic drug cisplatin by downregulating PD-L1 to activate NLRP3-mediated pyroptosis and inhibit cancer stem cell (CSC) properties, thereby sensitizing cisplatin-resistant gastric cancer cells to cisplatin (Li et al., 2021). LncRNA ADAMTS9-AS2 acts as a tumor suppressor in gastric cancer cells by activating NLRP3-mediated pyroptosis via miR-223-3p, thereby enhancing cisplatin sensitivity (Ren et al., 2020).

The dual role of NLRP3 in the development and treatment of gastric cancer could be due to its involvement in inflammatory infiltration during early cancer development, where NLRP3-mediated inflammation accelerates cancer progression. Conversely, during gastric cancer treatment, NLRP3-mediated pyroptosis can be exploited to induce cancer cell death, achieving anticancer effects.

## 9 Leukemia

### 9.1 Necroptosis and leukemia

Inhibitor of apoptosis proteins (IAPs) impede programmed cell death through various mechanisms and are associated with poor prognosis in acute myeloid leukemia (AML) (Tamm et al., 2000; Lück et al., 2011; Fulda and Vucic, 2012). The second mitochondria-derived activator of caspases (SMAC) can bind to and inactivate IAPs. Consequently, researchers have developed SMAC mimetics to induce necroptosis in apoptosis-resistant AML cells. Studies demonstrate that SMAC mimetics enhance AML cell sensitivity to apoptosis more effectively than birinapant; the combination with the caspase inhibitor IDN-6556, which blocks caspase-8 activity, can potentiate the anti-cancer effects of birinapant by triggering necroptosis (Brumatti et al., 2016) Another SMAC mimetic, BV6, mediates necroptosis by regulating TNF-α and its downstream RIPK1-RIPK3-MLKL signaling pathway. Moreover, BV6 induces necroptosis in apoptosis-resistant patient-derived AML cells (Safferthal et al., 2017). When used in conjunction with standard chemotherapeutic agents such as cytarabine, azacitidine, and decitabine, BV6 promotes necroptosis in resistant AML cells, thereby enhancing their eradication. Several clinical studies on SMAC mimetics for leukemia treatment are underway, offering promising new strategies and potential drug discoveries for combating leukemia.

### 9.2 Pyroptosis and leukemia

A study (Wang et al., 2017) demonstrated that chemotherapy drugs can induce pyroptosis in leukemia cells by activating caspase-3, which cleaves GSDME, leading to the formation of pores in the cell membrane and subsequent cell death. This process not only eliminates leukemia cells but also enhances the anti-leukemic effects of chemotherapy through the release of pro-inflammatory cytokines.

Another study (Urwanisch et al., 2021) further elucidated the role of the NLRP3 inflammasome in leukemia, showing that it mediates IL-1β release through both GSDMD-dependent and independent mechanisms. This dual pathway highlights the complex regulation of pyroptosis in leukemic cells and its impact on the inflammatory environment within tumors.

Zhang Z. et al. (2020) identified GSDME as a key player in anti-tumor immunity in leukemia. They found that GSDME activation induces pyroptosis in leukemia cells and enhances immune system recognition and attack on these cells. This suggests that targeting GSDME-mediated pyroptosis could be a promising therapeutic strategy to boost immune responses against leukemia.

Fang et al. (2020) detailed the mechanisms by which pyroptosis is activated in leukemia cells, primarily through the caspase-1 and caspase-4/5/11 pathways. The cleavage of GSDMD during pyroptosis results in pore formation in the cell membrane, leading to cell lysis and the release of pro-inflammatory cytokines like IL-1β and IL-18. This process can effectively eliminate leukemia cells and stimulate an anti-tumor immune response. However, they also highlights the potential adverse effects of chronic inflammation associated with pyroptosis, which may contribute to tumor progression and resistance. Therefore, while pyroptosis offers a promising mechanism for anti-leukemic therapy, further research is needed to optimize therapeutic strategies that maximize its benefits and mitigate its inflammatory risks.

## 10 Other cancers

In addition to the common cancers mentioned above, research indicates that necroptosis and its key targets are involved in the development and progression of various other cancers. For example, reduced expression of RIPK3 has been observed in head and neck squamous cell carcinoma (McCormick et al., 2016), melanoma (Geserick et al., 2015), primary malignant mesothelioma (Tan et al., 2021), and prostate cancer (Wang KJ. et al., 2020). In these cancers, decreased RIPK3 expression is associated with cancer progression, metastasis, and shortened overall survival (Nugues et al., 2014; Geserick et al., 2015; Conev et al., 2019). Similarly, reduced MLKL expression has been noted in ovarian cancer (He et al., 2013), cervical squamous cell carcinoma (Ruan et al., 2015), colon cancer (Li et al., 2017), and in patients with early resected pancreatic adenocarcinoma, correlating with decreased overall survival (Colbert et al., 2013). Furthermore, downregulation of RIPK1 expression in head and neck squamous cell carcinoma has been closely linked to disease progression (McCormick et al., 2016). These findings suggest that necroptosis, akin to apoptosis, serves as a natural barrier against cancer development.

However, in certain cancers, the expression of necroptosis and its key targets is elevated. In pancreatic ductal adenocarcinoma, high levels of RIPK1, RIPK3, and MLKL are observed (Seifert et al., 2016; Wang W. et al., 2018; Ando et al., 2020). Knockout of RIPK3 or inhibition of RIPK1 can prevent the further development of KrasG12D-induced pancreatic ductal adenocarcinoma in mice (Seifert et al., 2016), and gemcitabine chemotherapy further increases the expression of RIPK1 and RIPK3 in cancer tissues. Elevated RIPK1 expression is also associated with poor prognosis in glioblastoma patients (Park et al., 2009), and high levels of phosphorylated MLKL correlate with poor prognosis and reduced overall survival in esophageal cancer patients (Liu et al., 2016). Additionally, studies have shown that RIPK1 expression is significantly elevated in human lung cancer samples and mouse lung tumor models, suggesting its role as a critical target in cancer induction (Wang et al., 2013). Recent research has found that dabrafenib, a drug used for treating melanoma, inhibits MLKL phosphorylation by disrupting the interaction between RIPK3 and MLKL (Li et al., 2014).

## 11 Emerging therapeutic strategies and prognostic tools in pyroptosis-driven cancer treatment

Recent advances in cancer research have underscored the significance of pyroptosis-related gene (PRG) signatures as promising prognostic tools and biomarkers across various cancer types. For instance, in breast cancer, 4 PRGs (GPX4, GSDMD, GSDMC, and IL18) were found to predict survival outcomes and the immune landscape of the tumor. This signature was linked to immune cell infiltration and tumor progression, making it a valuable tool for assessing patient prognosis and guiding therapeutic decisions (Gong et al., 2022).

Similarly, in HCC, a study identified a set of 52 differentially expressed PRGs, including key genes like IL-1β, NLRP3, and TP53, which were associated with patient survival and response to treatment. The genetic mutations and expression variations of these PRGs were analyzed, revealing their potential as prognostic markers (Fang et al., 2022). Moreover, the model not only predicted patient survival but also correlated with immune cell infiltration levels in the tumor immune microenvironment (TIME).

Pyroptosis plays a critical role in shaping the TIME. The release of inflammatory cytokines and DAMPs during pyroptosis not only leads to the death of tumor cells but also recruits and activates various immune cells, including macrophages, dendritic cells, and cytotoxic T lymphocytes (Chen Q. et al., 2024; Li and Jiang, 2023; Hu et al., 2024). This inflammatory response can enhance the immunogenicity of the tumor, leading to a more robust anti-tumor immune response. For instance, the activation of the NLRP3 inflammasome in tumor-associated macrophages and the subsequent pyroptosis can shift the immune balance from an immunosuppressive to an immunostimulatory state, potentially improving the efficacy of immunotherapies such as checkpoint inhibitors (Sun et al., 2024).

The ability of pyroptosis to modulate the TIME has significant implications for cancer immunotherapy. By enhancing the infiltration and activation of cytotoxic T lymphocytes and other effector immune cells, pyroptosis can potentially overcome the immunosuppressive barriers often encountered in the tumor microenvironment. Furthermore, the induction of pyroptosis and necroptosis in tumor cells may synergize with existing immunotherapeutic approaches, such as PD-1/PD-L1 blockade, by increasing the visibility of the tumor to the immune system (Liang et al., 2024; Yu et al., 2024). Targeting pyroptosis pathways, therefore, holds promise not only as a direct anti-cancer strategy but also as a means to enhance the effectiveness of immunotherapies.

Nanomaterials have emerged as promising tools in cancer therapy due to their unique physicochemical properties, such as high surface area and tunable surface chemistry, which enable them to interact with biological systems in novel ways. Recent research highlights how these materials, such as mannose-doped metal-organic frameworks, can trigger pyroptosis through pathways like the PERK pathway. For instance, Fe₃O₄@NH₂-MIL-100 nanoparticles doped with mannose have been shown to induce pyroptosis in tumor cells effectively. This effect is achieved by enhancing the activation of GSDMD, leading to cancer cell death (Jin et al., 2023). Another study designed fluorescent nanoprobes composed of AS1411 aptamer and nucleus-targeting peptide on gold nanoparticles to effectively capture and track the nucleolin distribution and expression during pyroptosis triggered by electrical stimulation, which hold great potential for cellular studies of cancer-related diseases (Kong et al., 2024). However, increased biomedical applications of nanomaterials raise considerable attention concerning their toxicological effects. Mesoporous silica nanoparticles (MSN) could trigger liver inflammation and hepatocyte pyroptosis through NLRP3 inflammasome activation, which was caused by MSN-induced ROS generation (Zhang et al., 2018).

The integration of pyroptosis-inducing agents with established cancer therapies, such as chemotherapy and immunotherapy, has shown promising potential in enhancing therapeutic outcomes.

### 11.1 Combining pyroptosis-inducing agents with chemotherapy

Chemotherapy is a cornerstone of cancer treatment, but resistance to chemotherapeutic agents remains a significant challenge. Recent studies suggest that inducing pyroptosis in conjunction with chemotherapy could overcome this resistance. For example, Diosbulbin-B effectively increases the sensitivity of gastric cancer cells to the chemotherapeutic drug cisplatin by downregulating PD-L1 to activate NLRP3-mediated pyroptosis (Li et al., 2021). This is particularly relevant in chemoresistant cancers, where inducing pyroptosis can overcome resistance mechanisms and improve treatment outcomes.

### 11.2 Combining pyroptosis-inducing agents with immunotherapy

The integration of pyroptosis-inducing agents with immunotherapy represents another promising avenue. Pyroptosis can lead to the release of damage-associated DAMPs and pro-inflammatory cytokines, which can potentiate anti-tumor immune responses. The use of immune checkpoint inhibitors, such as PD-1/PD-L1 blockers, in combination with pyroptosis-inducing agents, has been proposed to enhance the recruitment and activation of immune cells within the tumor microenvironment (Yu et al., 2024). This combination could potentially turn “cold” tumors, which are immunologically inactive, into “hot” tumors, making them more responsive to immunotherapy (Chen X. et al., 2024).

The combination of pyroptosis-inducing agents with other therapeutic modalities holds great promise for improving cancer treatment outcomes. Future research should focus on optimizing the timing, dosage, and sequence of these combinations to maximize their therapeutic efficacy while minimizing potential adverse effects. Additionally, exploring the molecular mechanisms underlying the synergistic interactions between pyroptosis and other treatments will be crucial for the development of novel therapeutic strategies.

## 12 Discussion and conclusion

Existing anticancer drugs primarily aim to induce apoptosis in cancer cells; however, cancer cells frequently develop resistance to apoptosis, leading to drug resistance. Consequently, bypassing apoptotic pathways to induce cancer cell death is crucial. Concurrently, an increasing body of research demonstrates that necroptosis and pyroptosis play vital roles in cancer progression, metastasis, prognosis, and immunosurveillance. Inducing necroptosis or pyroptosis through pharmacological means has emerged as a novel therapeutic approach to circumvent apoptosis resistance in cancer treatment. Nevertheless, the precise roles of these processes in cancer development remain contentious.

Studies indicate that RIPK3 is underexpressed in two-thirds of cancer cell lines, suggesting that the inhibition or absence of the necroptosis pathway may facilitate cancer progression (Graham, 2014). Pharmacological restoration or activation of necroptosis has been shown to effectively kill cancer cells. Conversely, some studies reveal that proteins in the necroptosis pathway are overexpressed in certain cancers, and activating necroptosis can lead to cancer cell metastasis and immunosuppression (Dupaul-Chicoine et al., 2010; Kim et al., 2013; Semper et al., 2014). Pyroptosis presents a similar dichotomy: some studies suggest that activation of inflammasomes or pyroptosis pathways promotes tumor microenvironment formation and accelerates cancer progression, while others demonstrate that drugs can induce cancer cell death by activating these pathways to achieve anticancer effects.

The “dual roles” of necroptosis and pyroptosis in cancer are not entirely contradictory. On one hand, necroptosis and pyroptosis can induce a robust immune response by releasing DAMPs and various immunomodulatory cytokines, thereby activating dendritic cells and enhancing antitumor immunity. This can effectively address tumor resistance, especially in cancers resistant to apoptosis, by inducing cancer cell death through programmed cell death pathways. On the other hand, as inflammatory programmed cell death modes, necroptosis and pyroptosis are accompanied by elevated inflammation levels, which significantly promote tumorigenesis and progression. Inflammation may enhance tumor angiogenesis, increase cancer invasiveness, and create an immunosuppressive tumor microenvironment. Early anti-inflammatory treatment in tumors can effectively inhibit tumor progression and malignancy transformation (Coussens and Werb, 2002).

Moreover, although numerous compounds and drugs have been identified that can induce inflammatory programmed cell death and show potential anticancer activity, most studies remain limited to *in vitro* experiments or animal models. The feasibility of these compounds as anticancer drugs requires further evaluation *in vivo* and in clinical trials. Additionally, while drug-induced inflammatory programmed cell death can effectively kill tumor cells, it remains unclear whether this will exacerbate inflammation in adjacent tissues and normal tissues, accelerate the formation of the tumor microenvironment, and promote the transformation of normal tissue into cancerous tissue. Therefore, improving the “target specificity” of such drugs to cancer tissues and reducing their effects on normal tissues is crucial. In summary, inflammatory programmed cell death is closely associated with the development and progression of various cancers. Further research in this area holds promise for the development of novel anticancer drugs.

## 13 Summary

### 13.1 Beneficial aspects of necroptosis and pyroptosis in cancer treatment

1. Cancer cell elimination: Both necroptosis and pyroptosis are forms of programmed cell death that lead to the destruction of cancer cells. They can serve as alternative mechanisms to apoptosis, especially in cancer cells that have developed resistance to apoptotic pathways.
2. Immune system activation: The inflammatory response induced by necroptosis and pyroptosis can stimulate the immune system to recognize and attack cancer cells. The release of DAMPs and cytokines during these processes can enhance the anti-tumor immune response.
3. Overcoming drug resistance: Many cancer therapies aim to induce apoptosis in cancer cells. However, some cancer cells develop resistance to apoptosis. Necroptosis and pyroptosis can bypass these resistance mechanisms, providing an effective way to kill apoptosis-resistant cancer cells.
4. Synergistic Effects with Chemotherapy: Compounds that induce necroptosis or pyroptosis can be used in combination with traditional chemotherapeutic agents to enhance their efficacy. This combination can lead to improved outcomes in cancer treatment by targeting cancer cells through multiple pathways.

### 13.2 Detrimental aspects of necroptosis and pyroptosis in cancer treatment

1. Chronic inflammation: The inflammatory response triggered by necroptosis and pyroptosis, while beneficial in the short term, can lead to chronic inflammation if not properly regulated. Chronic inflammation is a known risk factor for cancer development and progression.
2. Damage to surrounding tissue: The inflammatory nature of necroptosis and pyroptosis can cause collateral damage to surrounding healthy tissues. This non-specific damage can result in adverse side effects and limit the therapeutic window of treatments that induce these forms of cell death.
